# Randomized Trial Replication Using Observational Data for Comparative Effectiveness of Secukinumab and Ustekinumab in Psoriasis: A Study From the British Association of Dermatologists Biologics and Immunomodulators Register

**DOI:** 10.1001/jamadermatol.2020.4202

**Published:** 2020-12-02

**Authors:** Zenas Z. N. Yiu, Kayleigh J. Mason, Philip J. Hampton, Nick J. Reynolds, Catherine H. Smith, Mark Lunt, Christopher E. M. Griffiths, Richard B. Warren

**Affiliations:** 1 Centre for Dermatology Research, Salford Royal NHS Foundation Trust, The University of Manchester, Manchester Academic Health Science Centre, National Institute for Health Research (NIHR) Manchester Biomedical Research Centre, Manchester, United Kingdom; 2 Institute of Translational and Clinical Medicine, Newcastle University Medical School, Newcastle upon Tyne, United Kingdom; 3 Department of Dermatology, Royal Victoria Infirmary and NIHR Newcastle Biomedical Research Centre, Newcastle Hospitals NHS Foundation Trust, Newcastle upon Tyne, United Kingdom; 4 St John’s Institute of Dermatology, Guy’s and St Thomas’ NHS Foundation Trust, London, United Kingdom; 5 Versus Arthritis Centre for Epidemiology, The University of Manchester, Manchester, United Kingdom

## Abstract

**Importance:**

Treatments for psoriasis may be less effective in everyday practice than in clinical trials. Emulating a target trial using data from the British Association of Dermatologists Biologics and Immunomodulators Register (BADBIR) can provide treatment effect estimates that are robust and can inform both clinicians and regulatory bodies.

**Objectives:**

To assess the comparative effectiveness of ustekinumab and secukinumab in patients with psoriasis, and to test whether the relative effectiveness estimate of the CLEAR trial, a randomized clinical trial that compared secukinumab with ustekinumab for psoriasis, can be replicated.

**Design, Setting, and Participants:**

This comparative effectiveness research study used a target trial emulation approach and was performed between November 2007 and August 2019. Data were obtained from BADBIR, a multicenter longitudinal pharmacovigilance register of patients with moderate to severe psoriasis in the United Kingdom and Republic of Ireland. Participants had chronic plaque psoriasis, were 18 years or older, and had at least 1 record of a Psoriasis Area and Severity Index (PASI) of 12 or higher before their initiation to secukinumab or ustekinumab. Propensity score (PS) 1:1 matched analysis and inverse probability treatment weighted analysis were performed.

**Main Outcomes and Measures:**

The primary outcomes were the risk ratio (RR) and the risk difference (RD) for achieving PASI of 2 or lower after 12 months of therapy for secukinumab compared with ustekinumab. Methods to account for missing outcome data were complete case analysis, nonresponder imputation, last observation carried forward, inverse probability of censoring weighting, and multiple imputation. Regulatory and estimate agreement metrics were used to benchmark the effect estimates in this study against those in the CLEAR trial.

**Results:**

A total of 1231 patients were included in the analysis, with 917 receiving ustekinumab and 314 receiving secukinumab. Secukinumab was superior to ustekinumab in all analyses, except under the nonresponder imputation method, in the proportion of participants achieving a PASI of 2 or lower (PS-weighted complete case analysis: RR, 1.28 [95% CI, 1.06-1.55]; RD, 11.9% [1.6-22.1]). All analyses, except for nonresponder imputation, reached regulatory agreement in both PS-matching and PS-weighted analyses.

**Conclusions and Relevance:**

This comparative effectiveness study found that secukinumab resulted in more patients achieving a PASI of 2 or lower after 12 months of therapy compared with ustekinumab in patients with psoriasis. Target trial emulation in this study resulted in regulatory and estimate agreement with the CLEAR randomized clinical trial; further such studies may help fill the evidence gap when comparing other systemic therapies for psoriasis.

## Key Points

**Question:**

What is the effectiveness of secukinumab compared with ustekinumab for the treatment of psoriasis in an everyday clinical setting?

**Findings:**

In this comparative effectiveness research study of 1231 patients receiving either secukinumab or ustekinumab for psoriasis, both drugs had lower treatment effectiveness in a real-world clinical setting than in a trial setting. Secukinumab had superior effectiveness compared with ustekinumab, and the estimate of this relative effect using observational data met regulatory and estimate agreement with trial data.

**Meaning:**

Results of this study found a gap between the efficacy of biologic therapies in an idealized trial setting and the effectiveness of biologic therapies in the real-world clinical setting in the treatment of psoriasis; however, a target trial emulation approach can provide robust estimates of relative effectiveness that can be used for clinical and regulatory decision-making.

## Teaser

This comparative effectiveness research study examines observational data from the British Association of Dermatologists Biologics and Immunomodulators Register and compares them with clinical trial data to determine the effectiveness of 2 psoriasis medications.

## Introduction

Evidence of the comparative effectiveness of systemic treatments for psoriasis is important to inform the treatment choice of patients and clinicians. Randomized clinical trials (RCTs) that compare 2 agents are the criterion standard level of evidence for treatment efficacy. However, current RCTs in psoriasis have several limitations. First, little incentive exists in the pharmaceutical industry to perform head-to-head studies unless drug manufacturers are confident that they will be able to demonstrate the superiority of their product. Second, evidence of the comparative efficacy of nonbiologic systemic therapies is lacking.^1^ Third, RCTs do not fully reflect the outcomes observed in real-world settings.^2,3^ Comparative effectiveness studies of systemic therapies for psoriasis that use observational data can provide findings to inform clinical evidence in the absence of RCT results. Such data may also help to quantify the efficacy-effectiveness gap, where efficacy is the performance of treatments under ideal conditions and effectiveness is the performance of treatments in a clinical setting, between trial and real-world outcomes if trial data are available.

Comparative effectiveness studies can be affected by several sources of bias, including, for example, selection, confounding, and immortal time bias. A method to avoid these biases is to explicitly characterize a target trial (ie, the ideal RCT that would be conducted if feasible) to provide a structured approach to guide the analysis and thereby minimize bias.^4,5,6^ This approach includes outlining the key aspects of the target trial, such as the eligibility criteria, treatment strategies, assignment procedure, follow-up period, and causal contrasts of interest, and then applying this design as much as possible to the observational data set.

We performed a benchmarking study to compare the effectiveness of secukinumab (Cosentyx) with that of ustekinumab (Stelara) using data from the British Association of Dermatologists Biologics and Immunomodulators Register (BADBIR) by emulating a pragmatic RCT. The relevant corresponding RCT was the CLEAR (A 52-week, Multicenter, Randomized, Double-blind Study of Subcutaneous Secukinumab to Demonstrate Efficacy as Assessed by Psoriasis Area and Severity Index at 16 Weeks of Treatment Compared to Ustekinumab and to Assess Long-term Safety, Tolerability and Efficacy in Subjects With Moderate to Severe Plaque Psoriasis) trial,^7,8^ a head-to-head comparison between secukinumab and ustekinumab. Our objective was to test 2 hypotheses: (1) the real-world effectiveness of both therapies in BADBIR was lower than that reported in the CLEAR trial, and (2) the relative effectiveness between the therapies documented in BADBIR in an emulated pragmatic RCT would be similar to their relative efficacy in an explanatory RCT (CLEAR trial). We used the agreement metrics described in an article by Franklin et al^6^ to evaluate whether we were successful in replicating the relative effect estimates of the CLEAR trial. These metrics were regulatory agreement, which indicates whether the study replicates the direction and statistical significance of the RCT findings; estimate agreement, which indicates whether the study effect estimate lies within the 95% CI of the effect estimate in the RCT; and the magnitude and direction of the differences between the study and RCT results, as measured by the standardized difference.

## Methods

This comparative effectiveness research study was performed between November 2007 and August 2019. BADBIR was approved in 2007 by the National Health Service Research Ethics Committee North West England. All participants in the registry gave written informed consent. This approval extends to the present study. We followed the Strengthening the Reporting of Observational Studies in Epidemiology (STROBE) reporting guideline.

### Data Source and Eligibility Criteria

BADBIR is a large registry of patients with moderate to severe psoriasis in the UK and Republic of Ireland who were receiving either a traditional systemic therapy, a new oral small molecule systemic therapy, or a biologic therapy. Established in September 2007 as a pharmacovigilance register, BADBIR contains detailed information on the disease characteristics, severity, and comorbid conditions of patients collected at baseline entry into the register. Subsequently, data, including disease severity and any adverse events (AEs), are collected every 6 months for the first 3 years and then annually thereafter. The study design and baseline characteristics of patients recruited in BADBIR have been reported in detail.^9,10^ A data cutoff date of August 2019 was used in the present study. Details of the reference RCT, the CLEAR trial, are shown in the eAppendix in the Supplement.

Individuals eligible for the present study had chronic plaque psoriasis and were 18 years or older. Past use of any systemic therapy was allowed, with the exception of ustekinumab, secukinumab, or other biologic therapies that targeted the interleukin 17 and 23 pathways. Individuals needed to have at least 1 record of a Psoriasis Area and Severity Index (PASI) of 12 or higher prior to the initiation of the biologic under investigation. We restricted the drug initiation to dates on or after September 2013, when both secukinumab and ustekinumab were available to patients in the UK clinical setting, and before September 2018 to allow individuals time to complete 12 months of treatment. We placed no exclusions on the basis of comorbid conditions to maximize external validity.

### Treatment Strategies and Follow-up

Participants were receiving either ustekinumab or secukinumab. Any concomitant topical therapies were allowed, as were concomitant systemic treatments for psoriasis, which we adjusted for in the data analysis. Dosing strategies were established by the individual clinicians. Follow-up for each participant started on the date that ustekinumab or secukinumab was initiated. Demographic characteristics of each participant were documented on or before this start date. Follow-up ended at the occurrence of death, loss to follow-up, discontinuation of therapy, or 12-month outcome. The reasons for discontinuation of therapy (eg, ineffectiveness, AEs, or remission) were documented by the clinical research team.

### Outcomes

The primary outcome of this study was the difference in the proportion of participants who achieved a PASI of 2 or lower after 12 months of therapy between the 2 comparator cohorts. We performed sensitivity analyses using PASI 90 (ie, ≥90% improvement in PASI) as an alternative secondary outcome. Justification for choosing these outcomes are provided in the eAppendix in the Supplement. The AEs and serious AEs were coded using the Medical Dictionary for Regulatory Activities (MedDRA) terms and presented in a descriptive analysis.

### Statistical Analysis 

A propensity score (PS) was fitted using multivariable logistic regression to account for potential confounding between the secukinumab and ustekinumab cohorts. Two PS methods were used: (1) PS nearest-neighbor optimal 1:1 matching with a caliper of 0.05 was performed to identify the secukinumab and ustekinumab cohorts in the first analysis, and (2) inverse probability treatment weighting was used to balance the 2 cohorts in the second analysis. Details of the covariates within the PS and the methods used are described in the eAppendix in the Supplement.

Only eligible individuals with no missing baseline data for the potential confounders were included in the study. In the case of missing outcome data, the outcome was imputed as a nonresponder after 12 months of treatment if the treatment was discontinued before that time point because of ineffectiveness or if an AE for psoriasis flare was entered within 1 month of the cessation of therapy. The outcome was imputed as a responder after 12 months of treatment if the treatment was discontinued because of disease remission.

To investigate the implication of missing outcome data for the effect estimate, we used nonresponder imputation (NRI), last observation carried forward (LOCF), multiple imputation (MI), and inverse probability of censoring weighting (IPCW) to account for missing outcome data in the separate analyses for the intention-to-treat estimate (ie, analysis of participants according to the original treatment assignment) and the complete case analysis (CCA) for the per-protocol estimate (ie, analysis of participants according to the completion of the assigned treatment). A summary of these methods is presented in the eAppendix in the Supplement.

Generalized linear models were fitted with a log link for the relative risk ratio (RR) and an identity link for the risk difference (RD). Robust SEs were estimated, and matched sets were accounted for in the PS-matched analysis. We performed a sensitivity analysis to investigate the implication of weight truncation for the PS-weighted analysis. We used the aforementioned metrics (ie, regulatory agreement, estimate agreement, and standardized difference) to benchmark the effect estimates in this study with those in the CLEAR trial^7,8^ that compared the PASI 90 achievement between secukinumab and ustekinumab.

All data analyses were performed with Stata, version 16.1 (StataCorp LLC).

## Results

A total of 1231 patients were included in the analysis, with 917 receiving ustekinumab and 314 receiving secukinumab. Table 1 shows the baseline characteristics of the 2 cohorts, and the patient flow diagram is presented in eFigure 1 in the Supplement. The PASI at 12 months was recorded for 132 participants (42.0%) in the secukinumab cohort and 417 participants (45.5%) in the ustekinumab cohort (eFigure 1 in the Supplement).

**Table 1.** Baseline Characteristics of the Ustekinumab and Secukinumab Cohorts

Characteristic	No. (%)	
	Ustekinumab cohort (n = 917)	Secukinumab cohort (n = 314)
Age, median (IQR), y	45.0 (35.0-55.0)	46.0 (36.0-55.0)
Female sex	365 (39.8)	121 (38.5)
BMI	30.9 (26.6-36.5)	30.5 (26.7-35.1)
Weight, median (IQR), kg	92.0 (77.2-107.5)	89.4 (76.4-107.0)
Alcohol		
No documented alcohol intake	338 (36.9)	115 (36.6)
Lower-risk drinking (male: <21 U/wk; female: <14 U/wk)	470 (51.3)	160 (51.0)
Hazardous drinking (male: <50 U/wk; female: <35 U/wk)	96 (10.5)	36 (11.5)
Harmful drinking (male: ≥50 U/wk; female: ≥35 U/wk)	13 (1.4)	3 (1.0)
Smoking status		
Never	320 (34.9)	126 (40.1)
Previous	346 (37.7)	109 (34.7)
Current	251 (27.4)	79 (25.2)
Baseline PASI	16.3 (13.9-21.1)	17.6 (14.4-22.7)
Psoriatic arthritis	135 (14.7)	64 (20.4)
No. of previous biologic therapies		
None	770 (84.0)	261 (83.1)
1	103 (11.2)	33 (10.5)
2	33 (3.6)	16 (5.1)
≥3	11 (1.2)	4 (1.3)
No. of previous conventional therapies		
None	72 (7.9)	30 (9.6)
1	209 (22.8)	88 (28.0)
2	302 (32.9)	120 (38.2)
≥3	334 (36.4)	76 (24.2)
Previously treated with TNF inhibitors	144 (15.7)	50 (15.9)
Treated with a concomitant systemic therapy	118 (12.9)	15 (4.8)
No. of comorbid conditions		
None	262 (28.6)	108 (34.4)
1-2	463 (50.5)	152 (48.4)
3-4	152 (16.6)	45 (14.3)
≥5	40 (4.4)	9 (2.9)
Psoriasis		
Palmoplantar	189 (20.6)	58 (18.5)
Nail	488 (53.2)	174 (55.4)
Scalp	690 (75.2)	234 (74.5)
Depression	227 (24.8)	62 (19.7)
Working status		
Full-time	493 (53.8)	191 (60.8)
Part-time	111 (12.1)	35 (11.1)
Full-time in the home	30 (3.3)	13 (4.1)
Unemployed but seeking work	39 (4.3)	11 (3.5)
Not working because of disability/ill health	117 (12.8)	28 (8.9)
Student	22 (2.4)	6 (1.9)
Retired	105 (11.5)	30 (9.6)
Race/ethnicity		
White	816 (89.0)	289 (92.0)
Black	9 (1.0)	0
Asian	54 (5.9)	14 (4.5)
Other	38 (4.1)	11 (3.5)

Abbreviations: BMI, body mass index (calculated as weight in kilograms divided by height in meters squared); IQR, interquartile range; PASI, Psoriasis Area and Severity Index; TNF, tumor necrosis factor.

The numbers of participants in each cohort who experienced AEs and serious AEs are presented in eTable 1 in the Supplement, with few serious AEs reported within 12 months in both cohorts. Fewer than 5 AEs in the secukinumab and ustekinumab cohorts were coded with the MedDRA high-level term of *fungal infection disorders*. No AEs in the secukinumab cohort and 5 AEs in the ustekinumab cohort were coded with MedDRA high-level term of *noninfective colitis*.

The PS matching analysis included 622 participants, with 311 participants in each treatment cohort (eFigure 2 in the Supplement). Weight truncation did not improve the precision of the estimate materially and therefore was not implemented (eTable 2 in the Supplement). Both PS matching and PS weighting methods resulted in 2 balanced cohorts (eTables 3 and 4 in the Supplement).

The RRs (Figure) and RDs (eFigure 3 in the Supplement) that compared the proportion of participants who achieved a PASI of 2 or lower at 12 months in the secukinumab and ustekinumab cohorts, estimated from the generalized linear models using the missing outcome imputation methods, are presented in Table 2. Table 2 also shows the corresponding outcome measures from the CLEAR trial. The proportion of participants who achieved the outcome was lower in this study, regardless of the methods used for confounding adjustment or outcome imputation, compared with the CLEAR trial (Table 2).

**Figure.**
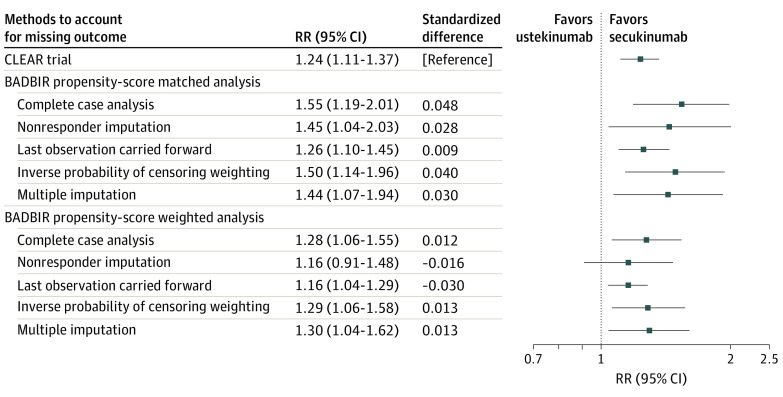
Forest Plot of the Risk Ratio (RR) Estimates for Participants Achieving Psoriasis Area and Severity Index of 2 or Lower at 12 Months

**Table 2.** Outcome of Absolute PASI of 2 or Lower at 12 Months for Secukinumab and Ustekinumab^a^

Data source and missing outcome analysis method	CLEAR trial NRI^b^	Present BADBIR study				
		CCA (n = 265 PS matched; n = 549 PS weighted)	NRI	LOCF (n = 559 PS matched; n = 1106 PS weighted)	IPCW	MI
PS-matched analysis (n = 622)						
Estimated proportion on secukinumab achieving PASI ≤2, % (95% CI)	74.9	59.8 (31.5 to 75.6)	24.7 (19.9 to 29.5)	68.6 (63.2 to 74.1)	60.3 (51.1 to 69.5)	59.6 (51.8 to 67.4)
Estimated proportion on ustekinumab achieving PASI ≤2, % (95% CI)	60.6	40.4 (21.8 to 64.2)	17.5 (13.0 to 22.0)	54.7 (48.4 to 61.0)	42.0 (32.7 to 51.3)	42.7 (32.3 to 53.0)
RR (95% CI)	1.24 (1.11 to 1.37)	1.55 (1.19 to 2.01)	1.45 (1.04 to 2.03)	1.26 (1.10 to 1.45)	1.50 (1.14 to 1.96)	1.44 (1.07 to 1.94)
Standardized difference between CLEAR and BADBIR studies for RR^c^	NA	0.048	0.028	0.009	0.040	0.030
RD, % (95% CI)	14.3 (7.2 to 21.1)	19.5 (0.1 to 31.6)	7.2 (0.4 to 13.9)	14.0 (5.9 to 22.1)	18.3 (5.5 to 31.1)	16.9 (3.3 to 30.6)
Standardized difference between CLEAR and BADBIR studies for RD^c^	NA	0.024	−0.047	−0.003	0.018	0.011
PS-weighted analysis (n = 1231)						
Estimated proportion on secukinumab achieving PASI ≤2, % (95% CI)	74.9	57.4 (48.3 to 66.4)	23.4 (18.5 to 28.2)	67.7 (62.0 to 73.4)	58.2 (48.5 to 67.9)	57.8 (48.5 to 67.1)
Estimated proportion on ustekinumab achieving PASI ≤2, % (95% CI)	60.6	45.5 (40.4 to 50.6)	20.4 (17.6 to 23.2)	58.6 (55.0 to 62.2)	45.9 (40.7 to 51.1)	44.7 (39.5 to 49.9)
RR (95% CI)	1.24 (1.11 to 1.37)	1.28 (1.06 to 1.55)	1.16 (0.91 to 1.48)	1.16 (1.04 to 1.29)	1.29 (1.06 to 1.58)	1.30 (1.04 to 1.62)
Standardized difference between CLEAR and BADBIR studies for RR^c^	NA	0.012	−0.016	−0.030	0.013	0.013
RD, % (95% CI)	14.3 (7.2 to 21.1)	11.9 (1.6 to 22.1)	2.9 (−2.6 to 8.5)	9.1 (2.4 to 15.8)	12.3 (1.4 to 23.2)	13.1 (1.3 to 24.9)
Standardized difference between CLEAR and BADBIR studies for RD^c^	NA	−0.012	−0.081	−0.034	−0.010	−0.006
Regulatory agreement^d^	NA	Yes	Yes (matched); No (weighted)	Yes	Yes	Yes
Estimate agreement^e^	NA	Yes (weighted); No (matched)	RR: yes (weighted); no (matched) RD: yes (matched); no (weighted)	Yes	RR: yes (weighted); no (matched) RD: yes	RR: yes (weighted); No (matched) RD: yes

Abbreviations: BADBIR, British Association of Dermatologists Biologics and Immunomodulators Register; CCA, complete case analysis; IPCW, inverse probability of censoring weighting; LOCF, last observation carried forward; MI, multiple imputation; NA, not applicable; NRI, nonresponder imputation; PASI, Psoriasis Area and Severity Index; PS, propensity score; RCT, randomized clinical trial; RD, risk difference; RR, risk ratio.

^a^ RR and RD were calculated using MedCalc.net.

^b^ Numbers from the PASI 90 nonresponder imputation outcome at week 52 in the CLEAR study.^7,8^

^c^ Standardized difference was calculated according to the methods in Franklin et al.^6^

^d^ Indicates whether the study replicates the direction and statistical significance of the CLEAR study^7,8^ finding.

^e^ Indicates whether the study treatment effect is within the 95% CI of the treatment effect estimate from the CLEAR study.^7,8^

All methods to account for missing outcome data, except NRI in the PS weighting, reached regulatory agreement for RRs and RDs (Table 2). However, a discrepancy was found regarding which method achieved estimate agreement. All methods achieved estimate agreement in the PS-weighted analysis, except for NRI for RD (Table 2). In contrast, CCA (RR, 1.55; 95% CI, 1.19-2.01), NRI (RR, 1.45; 95% CI, 1.04-2.03), IPCW (RR, 1.50; 95% CI, 1.14- 1.96), and MI (RR, 1.44; 95% CI, 1.07-1.94) all overestimated the RR in the PS-matched analysis, violating estimate agreement. The PS-matched analysis performed better in estimation of RD, reaching estimate agreement for all missing outcome imputation methods (CCA: 19.5% [95% CI, 0.1%-31.6%]; NRI: 7.2% [95% CI, 0.4%-13.9%]; IPCW: 18.3% [95% CI, 5.5%-31.1%]; LOCF: 14.0% [95% CI, 5.9%-22.1%]; MI: 16.9% [95% CI, 3.3%-30.6%]) (Table 2). The method that resulted in the lowest standardized difference for RR and RD between this study and the CLEAR trial was LOCF with PS matching (RR, 0.009; RD, −0.003). Secukinumab was superior to ustekinumab in all analyses, except under the nonresponder imputation method, in the proportion of participants achieving a PASI of 2 or lower (PS-weighted complete case analysis: RR, 1.28 [95% CI, 1.06-1.55]; RD, 11.9% [1.6-22.1]). The PS-weighted sensitivity analysis using PASI 90 as an alternative outcome gave similar results (eTable 5 in the Supplement).

## Discussion

To our knowledge, this study was the first to use a target trial approach in comparative effectiveness research of systemic therapies for psoriasis. Using this method, we showed that a robust analysis of data from BADBIR can yield comparative treatment effect estimates that replicate the estimates reported in an RCT and therefore can be reliable for clinical decision-making.

We found that NRI was not an appropriate missing outcome analysis method for the observational data. Using NRI was overly conservative and introduced nondifferential misclassification that biased the effect estimate toward the null and underestimated the effect size for each treatment cohort. The LOCF method produced the most precise results, but its estimate was less accurate when using PS weighting than the corresponding estimate in the CLEAR trial, shifting the effect estimate slightly toward the null. In addition, single imputation methods such as NRI and LOCF lead to an underestimation of SEs and uncertainty and may increase the likelihood of finding a false-positive result.^11^ We found that CCA, MI, and IPCW generated similar effect estimates and width of CIs using PS weighting.

### Insights for Clinical Practice

Taking the point estimate from CCA under PS weighting, we observed a 17.5% reduction for secukinumab and 15.1% reduction for ustekinumab between efficacy and effectiveness using the 12-month end point (Table 2). Patients and clinicians should, therefore, be informed that the probability of achieving a PASI of 2 or lower or PASI 90 was lower for both secukinumab and ustekinumab cohorts in the real world than the odds reported in the CLEAR trial, and secukinumab had statistically superior effectiveness compared with ustekinumab. When counseling patients about the likely outcome of biologic therapies on the basis of figures from clinical trials, clinicians should include a caveat that the real-world outcome may be around 15% lower than that found in clinical trials.

We have shown that the target trial method using observational data can lead to robust treatment effect estimates for psoriasis. As a result, we believe that clinicians can interpret other or future comparative effectiveness studies with a target trial framework with confidence and use this information in shared decision-making as an adjunct to data from RCTs.

Previous studies have shown that drug survival of secukinumab is similar to that of ustekinumab at year 1 when only the discontinuation because of ineffectiveness is taken into account.^12^ Thus, the results of this study suggest that drug survival alone cannot be a sensitive proxy for effectiveness.^13^

### Assumptions

An important bias to consider is that knowledge of the findings from the corresponding CLEAR trial may have altered the current study. To further test the robustness of this process, the method should be repeated using BADBIR data to conduct a comparative effectiveness study of drugs with corresponding, ongoing RCTs. Similar to other benchmarking studies, this study assumed that trial participation had no direct impact and that no unmeasured confounding was associated with the impact of trial participation.^14^

There are 2 assumptions with PS methods in observational data. The first is the strong ignorability of sample selection assumption, which assumes that no unobserved confounding leads to the differences in the distribution of potential outcomes for the 2 treatments.^15^ This assumption is unlikely to be completely true, but given that the effect estimate in this study closely replicated that in the CLEAR trial, it is unlikely that any unmeasured confounding has important implications. The second assumption is the stable unit treatment value assumption, which assumes that the treatment has the same implication for an individual regardless of how the individual came to be treated and that the implication of the treatment for the individual is independent of the treatment of other individuals.^16^ This assumption is associated with whether the study results can be generalized beyond the study population, and our ability to replicate the RR from the CLEAR trial suggests that this second assumption may also hold true.

This calibration study is a first step in demonstrating that real-world evidence can be analyzed in a way that tests other hypotheses of the comparative effectiveness of psoriasis treatments. We encourage researchers to test the robustness of this approach by conducting benchmarking replication studies using other psoriasis registries and a target trial framework. Although this approach cannot and should not take the place of the criterion standard of head-to-head biologics RCT, it has the potential to further such trials and may help fill the comparative effectiveness gap for systemic treatments of psoriasis. Such replication studies may provide more timely but similarly robust information to inform clinical decision-making.

### Strengths and Limitations 

We were able to perform this study because of the detailed baseline participant data captured in BADBIR, including important covariates from demographic data, disease severity, and specific site involvement, comorbid conditions, and concomitant treatments. Having such data allowed us to balance the 2 cohorts to minimize confounding. The target trial approach also necessitated a new user active-comparator study design, a robust design that leads to balanced patient baseline covariates between 2 treatment comparators.^17^ Unlike analyses that used routinely collected health care or insurance claims data, the present study faced little potential misclassification in the diagnosis of psoriasis and the specific biologic therapy the patient received. This analysis would not be possible if we used routinely collected health care or insurance claims data because their sources generally do not record disease severity along with commencement or subsequent claims of a drug.

The main limitation pertains to the amount of missing outcome data, with approximately 45% of the cohort having a recorded PASI at 12 months available for analysis (eFigures 1 and 2 in the Supplement). We believe that it is reassuring that the CCA and the MI analyses in the PS-weighted analysis using the entire cohort, which estimated the mean treatment effect in the whole population, gave RRs similar to those in the CLEAR RCT. This finding suggests that this missing mechanism of PASI at 12 months was either missing completely at random or missing at random; both situations can be accounted for using the analyses in this study. The other possibility was that the PASI was missing not at random but was missing similarly in both treatment cohorts and thus not exerting a substantial differential implication for the estimate of relative treatment effect. Because BADBIR does not require recording of data at 16 weeks, we were unable to use the primary end point of PASI 90 at week 16 in the CLEAR trial as the benchmark effect estimate. Although the AEs were reported herein for completeness, the comparative safety of secukinumab and ustekinumab should not be interpreted from this analysis; rather, an analysis that considers the potential confounders specific to the AE should be undertaken in a larger cohort.

## Conclusions

In this comparative effectiveness research study, treatment with secukinumab resulted in a higher proportion of patients reaching a PASI of 2 or lower after 12 months of therapy compared with the ustekinumab cohort in the BADBIR population, but an efficacy-effectiveness gap was found for both treatments. A target trial emulation approach can be used to perform comparative effectiveness studies in BADBIR with a high degree of agreement with a corresponding RCT for relative effectiveness of therapies for psoriasis. We suggest the use of this target trial framework in future comparative effectiveness studies that use data from BADBIR. Such research also should consider using PS weighting for confounding adjustment and either the CCA, MI, or IPCW method for imputing missing outcome data.

## Notes

Supplement. **eAppendix.** **eReferences.** **eTable 1.** Adverse Events and Serious Adverse Events as Coded by MedDRA System Organ Class in the Two Cohorts **eTable 2.** Distribution of the Inverse Probability Weights Used in the Analysis **eTable 3.** Baseline Characteristics of the Ustekinumab and Secukinumab Cohorts After Propensity Score Matching **eTable 4.** Baseline Characteristics of the Ustekinumab and Secukinumab Cohorts After Propensity Score Weighting **eTable 5.** Sensitivity Analyses Using the Alternative Outcomes of PASI 90 in the Propensity Weighted Cohort **eFigure 1.** Flow Chart Outlining Cohort Delineation in the Weighted Cohort Study **eFigure 2.** Flow Chart Outlining Cohort Delineation in the Matched Cohort Study **eFigure 3.** Forest Plot Summarizing the Risk Difference Estimates for the Proportion of Participants Achieving PASI ≤2 at 12 Months Comparing Secukinumab Against Ustekinumab Using the Two Propensity Score Methods and the Missing Outcome Analysis Methods
