# Supplementary material for: Randomized Trial Replication Using Observational Data for Comparative Effectiveness of Secukinumab and Ustekinumab in Psoriasis: A Study From the British Association of Dermatologists Biologics and Immunomodulators Register
Source: JAMA Dermatol. 2020 Dec 2;157(1):1–8. doi: 10.1001/jamadermatol.2020.4202 (PMC7711562; doi:10.1001/jamadermatol.2020.4202)
Supplement: Supplement. — eAppendix. eReferences. eTable 1. Adverse Events and Serious Adverse Events as Coded by MedDRA System Organ Class in the Two Cohorts eTable 2. Distribution of the Inverse Probability Weights Used in the Analysis eTable 3. Baseline Characteristics of the Ustekinumab and Secukinumab Cohorts After Propensity Score Matching eTable 4. Baseline Characteristics of the Ustekinumab and Secukinumab Cohorts After Propensity Score Weighting eTable 5. Sensitivity Analyses Using the Alternative Outcomes of PASI 90 in the Propensity Weighted Cohort eFigure 1. Flow Chart Outlining Cohort Delineation in the Weighted Cohort Study eFigure 2. Flow Chart Outlining Cohort Delineation in the Matched Cohort Study eFigure 3. Forest Plot Summarizing the Risk Difference Estimates for the Proportion of Participants Achieving PASI ≤2 at 12 Months Comparing Secukinumab Against Ustekinumab Using the Two Propensity Score Methods and the Missing Outcome Analysis Methods [file jamadermatol-e204202-s001.pdf]

Supplementary Online Content

Yiu ZZN, Mason KJ, Hampton PJ, et al; for the BADBIR Study Group: Randomized trial replication using observational data for comparative effectiveness of secukinumab and ustekinumab in psoriasis: a study from the British Association of Dermatologists Biologics and Immunomodulators Register. *JAMA Dermatol.* Published online December 2, 2020. doi: 10.1001/jamadermatol.2020.4202

eAppendix.

eReferences.

eTable 1. Adverse Events and Serious Adverse Events as Coded by MedDRA System Organ Class in the Two Cohorts

eTable 2. Distribution of the Inverse Probability Weights Used in the Analysis

eTable 3. Baseline Characteristics of the Ustekinumab and Secukinumab Cohorts After Propensity Score Matching

eTable 4. Baseline Characteristics of the Ustekinumab and Secukinumab Cohorts After Propensity Score Weighting

eTable 5. Sensitivity Analyses Using the Alternative Outcomes of PASI 90 in the Propensity Weighted Cohort

eFigure 1. Flow Chart Outlining Cohort Delineation in the Weighted Cohort Study

eFigure 2. Flow Chart Outlining Cohort Delineation in the Matched Cohort Study

eFigure 3. Forest Plot Summarizing the Risk Difference Estimates for the Proportion of Participants Achieving PASI ≤ 2 at 12 Months Comparing Secukinumab Against Ustekinumab Using the Two Propensity Score Methods and the Missing Outcome Analysis Methods

This supplementary material has been provided by the authors to give readers additional information about their work.

© 2020 Yiu ZZN et al. *JAMA Dermatology.*

eAppendix.

Reference trial

The CLEAR study was a phase IIIB randomized controlled trial[1] comparing secukinumab, an interleukin (IL)-17A inhibitor against ustekinumab, an IL-12/23 inhibitor that lasted 52 weeks. 676 participants were recruited into two 1:1 randomized groups to receive secukinumab (n=337) or ustekinumab (n=339) under the licensed recommended dosing regimen, with placebo injections given to patients on ustekinumab to maintain blinding. The primary endpoint was the percentage of participants achieving a 90% reduction in PASI (PASI 90) at week 16; a secondary endpoint was the percentage of participants achieving PASI 90 at week 52. Both non-responder imputation (NRI) and multiple imputation (MI) were used to impute for missing outcome data. The main inclusion criteria for CLEAR were age ≥18 years and Psoriasis Area and Severity Index (PASI) ≥12. The main exclusion criteria were non-plaque type psoriasis; previous exposure to biologic therapies that inhibited IL-17A (including IL-17 receptor A inhibitor), or IL-12/23; history of malignancy within 5 years; or any conditions that would preclude adherence to the trial protocol[1]. No concomitant use of systemic or topical psoriasis therapy was allowed.

Sample size calculation reference for the propensity score matched analysis

For an anticipated clinical difference of 15% higher proportion of participants achieving PASI ≤2 at 12 months in the secukinumab arm compared with the ustekinumab arm, which is taken from the secondary 1 year endpoint in the CLEAR study, assuming α=0.05, power of 0.90, and assuming ustekinumab achieving 60% PASI ≤2 and 1:1 group sizes, a total of 406 participants are required with 203 participants in each group.

Propensity score construction

Variables identified to be potential confounders between treatment choice and the outcome of PASI≤2 were: age; body mass index; weight; and psoriatic arthritis (PsA). Variables identified to be potentially associated with the outcome and hence essential to balance between the cohorts were: sex; alcohol intake; smoking intake; baseline PASI; biologic exposure status; number of previous conventional treatments; number of previous biologic treatments; number of comorbidities; ethnicity; work status; depression[2]; scalp psoriasis; palmoplantar psoriasis; and nail psoriasis[3]. These variables were included in the multivariable logistic regression to derive a propensity score (PS) for treatment with secukinumab after application of the eligibility criteria. The mean propensity score was 0.26 with a standard deviation of 0.08, and a range between 0.02 and 0.58.

An overview of PS methods is given in a paper by Peter Austin.[4] PS can be used to match; stratify; weight; and used for covariate adjustment to balance for covariates and therefore adjust for potential confounding. Matching and weighting were utilised in this study. We used a nearest neighbour optimal

© 2020 Yiu ZZN et al. *JAMA Dermatology.*

matching algorithm to produce matched pairs. Optimal matching selects matched samples with the smallest average absolute distance in propensity score across all matched pairs of a patient on secukinumab and a patient on ustekinumab. A caliper, i.e. the absolute difference allowed in the propensity scores of the matched pairs, of 0.05 was used. We used inverse probability treatment weighting, where a weighting on propensity score creates a pseudo-population in which the distribution of measured baseline covariates is independent of treatment assignment. A patient on secukinumab is given a weight of 1 / PS; a patient on ustekinumab is given a weight of 1 / (1 – PS). The distribution for the overall weights, calculated by the multiplication between the inverse probability treatment weighting and the inverse probability censoring weighting, is presented in Supplementary Table 2, along with the results from the sensitivity analysis on the impact of weight truncation.

Missing outcome imputation

Several methods were used to impute missing outcome data for the intention-to-treat estimate. In non-responder imputation, any participant who did not have a documented PASI at 12 months was classed being a non-responder. This is the most conservative method to impute outcome. In last observation carried forward, the last documented PASI from 16 weeks onwards was allowed to count as the participant's PASI at 12 months. Multiple imputation is a statistical method used to impute missing data. Several imputed datasets are generated with a predictive distribution based on the available background data of the participants. A model of interest is fitted in each dataset and combined to give an overall estimate. Twenty imputed datasets were used for the multiple imputation analysis. In the multiple imputation analysis in this manuscript, we used the baseline patient variables included in the PS in the model.

Inverse probability of censoring weighting (IPCW) corrects for missing outcome, or censoring, by giving extra weight to participants who have a documented PASI outcome at 12 months. Each participant is given a weight which is inversely proportional to the estimated probability of having no missing outcome. The IPCW was derived from a logistic regression model fitted with a dichotomous dependent variable of whether PASI was missing at 12 months, and the same independent variables as the covariates included in the PS. Weights for the two cohorts were calculated separately. We used a complete case analysis to calculate the per-protocol estimate. Detailed summaries of these methods are given in articles by Wood *et al*.[5], Pedersen *et al*.[6], and Rombach *et al*.[7].

Justification for using PASI ≤2 at 12 months as the primary outcome

Although a 90% reduction in PASI (PASI 90) is a widely used outcome in RCTs for biologic therapies in psoriasis, this may be problematic in observational studies and pragmatic RCTs. A traditional RCT for biologic therapies stipulates a washout period for many systemic therapies for psoriasis to meet a strict inclusion criterion for baseline disease severity, e.g. PASI ≥12. Real-world clinical practice and a pragmatic RCT approach, conversely, would allow for overlap of therapies and would not require a strict baseline PASI level immediately prior to commencement of treatment. The magnitude of the

© 2020 Yiu ZZN et al. *JAMA Dermatology.*

baseline PASI may determine whether a participant is able to achieve PASI 90, and a recent study suggested that PASI percentage change is not a suitable outcome for follow-up or to guide clinical practice[8]. Our previous work suggested that absolute PASI ≤2 was concordant with PASI 90, with PASI ≤2 assigning the same response status as PASI 90 in 89.7% of cases with a sensitivity of 96.9% and a specificity of 86.2%[9]. In this study population, PASI ≤2 assigns the same response status as PASI 90 in 94.2% of cases, with a sensitivity of 90.6% and a specificity of 97.8%.

The 12 month time-point was chosen as this is the closest available measured time-point in BADBIR to a secondary outcome time-point of the CLEAR study[1]. A window of 335 days (~11 months) to 395 days (~13 months) after drug initiation was allowed for the ascertainment of the 12 month outcomes. If there were two records of PASI within the time window, the closest value to 365 was taken for the 12 month outcome. For last observation carried forward (LOCF), any PASI measured from 16 weeks after start of treatment was eligible to be carried forward.

© 2020 Yiu ZZN et al. *JAMA Dermatology.*

eReferences

1. Blauvelt A, Reich K, Tsai TF, et al. Secukinumab is superior to ustekinumab in clearing skin of subjects with moderate-to-severe plaque psoriasis up to 1 year: Results from the CLEAR study. *J Am Acad Dermatol.* 2017;76(1):60-69 e69.
2. Macfarlane GJ, Pathan E, Jones GT, Dean LE. Predicting response to anti-TNFalpha therapy among patients with axial spondyloarthritis (axSpA): results from BSRBR-AS. *Rheumatology (Oxford).* 2020.
3. Warren RB, Marsden A, Tomenson B, et al. Identifying demographic, social and clinical predictors of biologic therapy effectiveness in psoriasis: a multicentre longitudinal cohort study. *Br J Dermatol.* 2019;180(5):1069-1076.
4. Austin PC. An Introduction to Propensity Score Methods for Reducing the Effects of Confounding in Observational Studies. *Multivariate Behav Res.* 2011;46(3):399-424.
5. Wood AM, White IR, Thompson SG. Are missing outcome data adequately handled? A review of published randomized controlled trials in major medical journals. *Clin Trials.* 2004;1(4):368-376.
6. Pedersen AB, Mikkelsen EM, Cronin-Fenton D, et al. Missing data and multiple imputation in clinical epidemiological research. *Clin Epidemiol.* 2017;9:157-166.
7. Rombach I, Jenkinson C, Gray AM, Murray DW, Rivero-Arias O. Comparison of statistical approaches for analyzing incomplete longitudinal patient-reported outcome data in randomized controlled trials. *Patient Relat Outcome Meas.* 2018;9:197-209.
8. Norlin JM, Nilsson K, Persson U, Schmitt-Egenolf M. Complete skin clearance and Psoriasis Area and Severity Index response rates in clinical practice: predictors, health-related quality of life improvements and implications for treatment goals. *Br J Dermatol.* 2020;182(4):965-973.
9. Mahil SK, Wilson N, Dand N, et al. Psoriasis treat to target: defining outcomes in psoriasis using data from a real-world, population-based cohort study (the British Association of Dermatologists Biologics and Immunomodulators Register, BADBIR). *Br J Dermatol.* 2019.

© 2020 Yiu ZZN et al. *JAMA Dermatology.*

eTable 1. Adverse Events and Serious Adverse Events as Coded by MedDRA System Organ Class in the Two Cohorts

	Propensity score weighted analysis				Propensity score matched analysis			
	Adverse events		Serious adverse events		Adverse events		Serious adverse events	
	Ustekinumab (n=917)	Secukinumab (n=314)	Ustekinumab	Secukinumab	Ustekinumab (n=311)	Secukinumab (n=311)	Ustekinumab	Secukinumab
**Blood and lymphatic system disorders**	5	0	0	0	<5	0	0	0
**Cardiac disorders**	7	6	<5	<5	<5	6	<5	<5
**Congenital, familial and genetic disorders**	<5	0	0	0	<5	0	0	0
**Ear and labyrinth disorders**	7	<5	0	<5	<5	<5	0	<5
**Endocrine disorders**	5	<5	0	<5	<5	<5	0	<5
**Eye disorders**	12	6	0	<5	5	6	0	<5
**Gastrointestinal disorders**	72	21	14	<5	24	21	5	<5
**General disorders and administration site conditions**	65	19	0	5	17	19	0	5
**Hepatobiliary disorders**	22	6	<5	<5	6	6	<5	<5
**Immune system disorders**	<5	<5	0	0	<5	<5	0	0
**Infections and infestations**	169	76	14	7	60	75	5	7
**Injury, poisoning, and procedural complications**	42	12	6	<5	11	12	<5	<5
**Investigations**	112	40	12	10	31	40	<5	10

© 2020 Yiu ZZN et al. *JAMA Dermatology.*

**Metabolism and nutrition disorders**	16	<5	<5	<5	5	6	<5	<5
**Musculoskeletal and connective tissue disorders**	97	17	<5	<5	30	17	<5	<5
**Neoplasms benign, malignant, and unspecified (incl cysts and polyps)**	16	<5	7	<5	5	<5	<5	<5
**Nervous system disorders**	47	18	<5	<5	18	18	0	<5
**Pregnancy, puerperium and perinatal conditions**	<5	0	<5	0	0	0	<5	0
**Psychiatric disorders**	36	5	5	<5	14	5	<5	<5
**Renal and urinary disorders**	10	5	<5	<5	<5	5	<5	<5
**Reproductive system and breast disorders**	9	6	0	0	<5	6	0	0
**Respiratory, thoracic, and mediastinal disorders**	58	23	<5	<5	14	23	<5	<5
**Skin and subcutaneous tissue disorders**	63	12	<5	<5	22	12	<5	<5
**Social circumstances**	<5	<5	0	0	<1	<5	0	0
**Surgical and medical procedures**	64	26	18	13	21	26	7	13
**Vascular disorders**	13	6	0	<5	7	6	0	<5

© 2020 Yiu ZZN et al. *JAMA Dermatology.*

eTable 2. Distribution of the Inverse Probability Weights Used in the Analysis

The progressive truncation of inverse probability weights is also presented, along with the changes to the relative risk ratio

**Truncation percentiles**	**Estimated weights**		**Relative risk ratio comparing secukinumab to ustekinumab (95% confidence intervals)**
	**Mean**	**Minimum/maximum**	
0, 100	0.999	0.289, 3.100	1.29 (1.06,1.58)
1, 99	0.997	0.449, 2.102	1.29 (1.06,1.57)
5, 95	0.989	0.645, 1.476	1.29 (1.07,1.56)
10, 90	0.980	0.726, 1.290	1.29 (1.07,1.56)

© 2020 Yiu ZZN et al. *JAMA Dermatology.*

eTable 3. Baseline Characteristics of the Ustekinumab and Secukinumab Cohorts After Propensity Score Matching

Baseline characteristic		Ustekinumab (N=311)	Secukinumab (N=311)	Standardised difference
Age (year) median, IQR		47.0 (35.0-55.0)	46.0 (36.0-55.0)	-0.064
Gender	Female	120 (38.6%)	118 (37.9%)	-0.013
BMI (kg/m2)		30.5 (26.7-36.1)	30.6 (26.7-35.2)	-0.036
Weight		91.0 (78.8-108.0)	89.6 (76.4-107.3)	-0.031
Alcohol intake	No documented alcohol intake	108 (34.7%)	113 (36.3%)	0.002
	Lower risk drink (<21U/WK M, <14U/WK F)	167 (53.7%)	160 (51.4%)	
	Hazardous drinking (<50U/WK M, < 35U/WK F)	33 (10.6%)	35 (11.3%)	
	Harmful drinking (>=50U/WK M, >=35U/WK F)	3 (1.0%)	3 (1.0%)	
Smoking status	Never Smoked	126 (40.5%)	125 (40.2%)	-0.016
	Previous Smoker	113 (36.3%)	108 (34.7%)	
	Current Smoker	72 (23.2%)	78 (25.1%)	
Baseline PASI		16.5 (14.1-21.7)	17.6 (14.4-22.6)	0.026
Psoriatic arthritis		63 (20.3%)	61 (19.6%)	-0.016
Number of previous biologic therapies	No previous biologic	256 (82.3%)	258 (83.0%)	-0.035
	1 previous biologics	33 (10.6%)	33 (10.6%)	
	2 previous biologics	14 (4.5%)	16 (5.1%)	
	3 or more previous biologics	8 (2.6%)	4 (1.3%)	
Number of previous conventional therapies	No previous conventionals	35 (11.3%)	28 (9.0%)	0.046
	1 previous conventional	89 (28.6%)	87 (28.0%)	
	2 previous conventionals	102 (32.8%)	120 (38.6%)	
	3 or more previous conventionals	85 (27.3%)	76 (24.4%)	

© 2020 Yiu ZZN et al. *JAMA Dermatology.*

Previously treated with TNF-α inhibitors*	No	256 (82.3%)	261 (83.9%)	/
Number of comorbidities	No comorbidity	99 (31.8%)	107 (34.4%)	-0.039
	1-2 comorbid conditions	159 (51.1%)	150 (48.2%)	
	3-4 comorbid conditions	44 (14.1%)	45 (14.5%)	
	5 or more comorbid conditions	9 (2.9%)	9 (2.9%)	
Palmoplantar psoriasis		57 (18.3%)	57 (18.3%)	0.000
Nail psoriasis		163 (52.4%)	172 (55.3%)	0.058
Scalp psoriasis		240 (77.2%)	232 (74.6%)	-0.060
Depression		55 (17.7%)	62 (19.9%)	0.030
Work status	Working full time	193 (62.1%)	190 (61.1%)	0.006
	Working part time	34 (10.9%)	34 (10.9%)	
	Working full time in the home	10 (3.2%)	12 (3.9%)	
	Unemployed but seeking work	6 (1.9%)	11 (3.5%)	
	Not working due to disability / ill health	32 (10.3%)	28 (9.0%)	
	Student	7 (2.3%)	6 (1.9%)	
	Retired	29 (9.3%)	30 (9.6%)	
Ethnicity	White	279 (89.7%)	286 (92.0%)	-0.055
	Black	1 (0.3%)	0 (0.0%)	
	Asian	21 (6.8%)	14 (4.5%)	
	Other	10 (3.2%)	11 (3.5%)	
Treatment with concomitant systemic therapy*		41 (13.2%)	14 (4.5%)	/

*Not included in the estimation of the propensity score as decision regarding treatment with concomitant systemic therapy occurs after treatment allocation.

© 2020 Yiu ZZN et al. *JAMA Dermatology.*

eTable 4. Baseline Characteristics of the Ustekinumab and Secukinumab Cohorts After Propensity Score Weighting

Baseline characteristic		Ustekinumab (n=917)	Secukinumab (n=314)	Standardised difference
Age (year)		45.3	45.5	0.015
Gender	Female	39.3%	38.2%	-0.024
BMI (kg/m2)		31.9	31.8	-0.008
Weight		93.7	93.6	-0.006
Alcohol intake	Units per week	7.5	7.9	0.020
Smoking status	Cigarettes per day	3.3	3.3	-0.010
Baseline PASI		18.5	18.5	-0.005
Psoriatic arthritis		16.2%	16.7%	0.013
Number of previous biologic therapies		0.2	0.2	0.023
Number of previous conventional therapies		2.2	2.1	-0.029
Number of comorbidities		1.5	1.5	0.001
Palmoplantar psoriasis		20.1%	20.1%	0.000
Nail psoriasis		53.7%	52.9%	-0.016
Scalp psoriasis		75.1%	74.7%	-0.010
Depression		23.0%	24.0%	0.005
Work status	Working full time	55.4%	57.3%	0.038
	Working part time	12.3%	10.1%	-0.069
	Working full time in the home	3.2%	3.6%	0.025
	Unemployed but seeking work	4.0%	4.3%	0.015
	Not working due to disability / ill health	12.1%	10.2%	-0.060
	Student	2.7%	2.2%	0.034
	Retired	11.7%	10.8%	0.029
Ethnicity	White	88.8%	89.2%	0.013
	Black	1.1%	0.7%	-0.047
	Asian	5.7%	6.2%	0.020
	Other	4.3%	3.9%	-0.022

© 2020 Yiu ZZN et al. *JAMA Dermatology.*

eTable 5. Sensitivity Analyses Using the Alternative Outcomes of PASI 90 in the Propensity Weighted Cohort

**Data source**		**CLEAR study**	**BADBIR**				
**Missing outcome analysis method**		**Non-responder imputation**	**Complete case analysis**	**Non-responder imputation**	**Last observation carried forward**	**Inverse probability of censoring weighting**	**Multiple imputation**
**Propensity score weighted analysis – PASI 90**	**Estimated proportion on secukinumab achieving PASI 90**	74.9%	54.0% (44.9 – 63.1)	21.8% (17.2 – 26.5)	62.9% (56.9 – 68.8)	53.9% (45.2 – 62.5)	54.0% (44.9-63.1)
	**Estimated proportion on ustekinumab achieving PASI 90**	60.6%	43.4% (38.3 – 48.5)	19.5% (16.8 – 22.3)	54.3% (50.6 – 57.9)	43.1 % (35.7-50.6)	43.4% (38.3 – 48.5)
	**Risk ratio (RR)**	1.24 (1.11 - 1.37)	1.27 (1.04 – 1.56)	1.14 (0.88 -1.47)	1.17 (1.04 – 1.31)	1.24 (1.00 – 1.55)	1.27 (1.04 – 1.56)
	**Risk difference (RD)**	14.3% (7.2 – 21.1)	10.6% (0.3 – 20.9)	2.3% (-3.1 – 7.7)	8.6% (1.7 – 15.5)	9.6% (-1.5 – 20.7)	10.6% (2.8 – 20.9)
	#**Regulatory agreement**	/	Y	N	Y	Y (RR) N (RD)	Y
	**$Estimate agreement**	/	Y	Y (RR) N (RD)	Y	Y	Y

*Risk ratio and risk difference calculated using MedCalc.net; numbers taken from the CLEAR study secondary outcome at week 52.

#Regulatory agreement – study replicates direction and statistical significance of the randomized controlled trial finding; $Estimate agreement – study treatment effect lies within the 95% CI for treatment effect estimate from the trial

© 2020 Yiu ZZN et al. *JAMA Dermatology.*

eFigure 1. Flow Chart Outlining Cohort Delineation in the Weighted Cohort Study

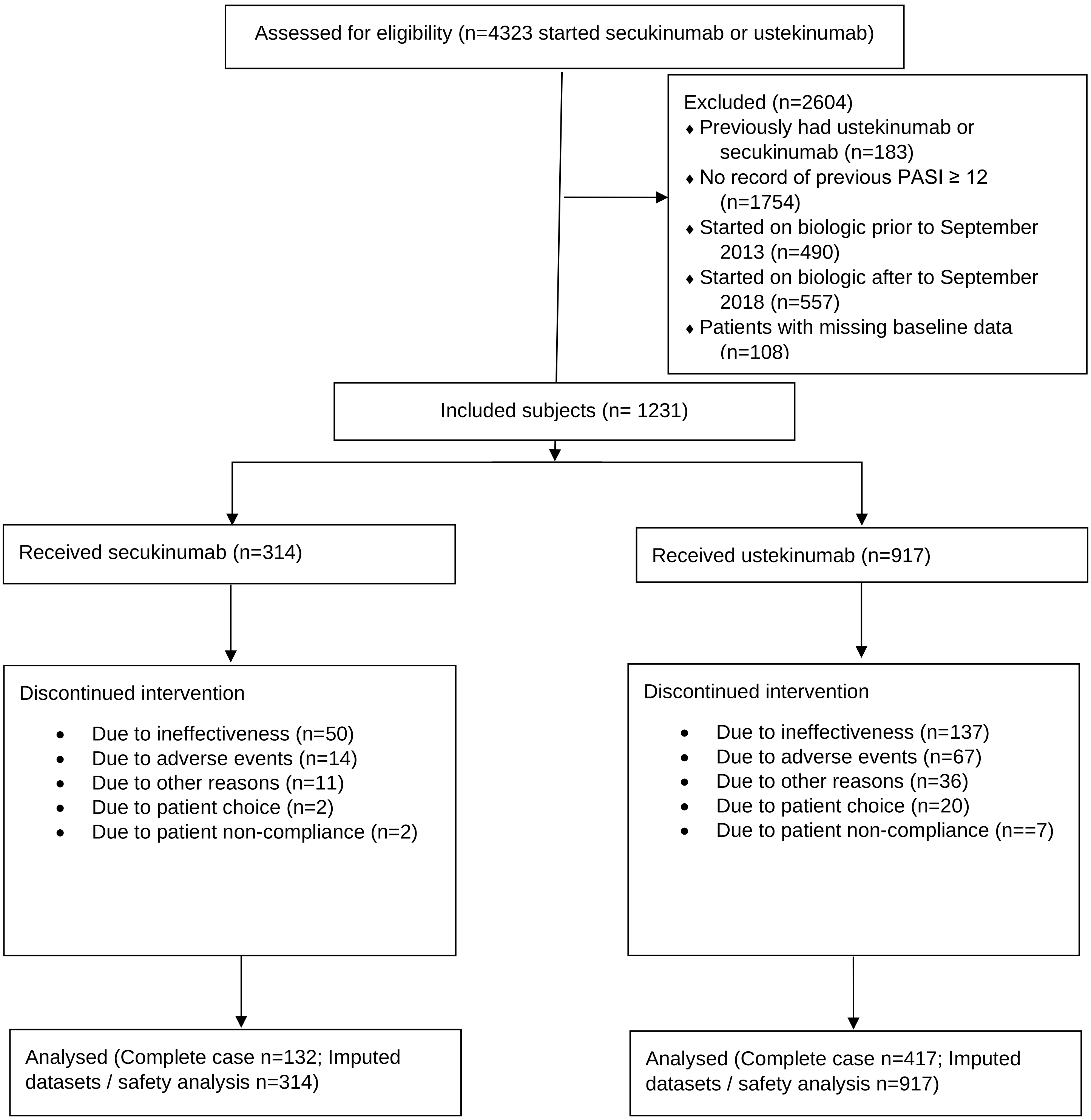

© 2020 Yiu ZZN et al. *JAMA Dermatology.*

eFigure 2. Flow Chart Outlining Cohort Delineation in the Matched Cohort Study

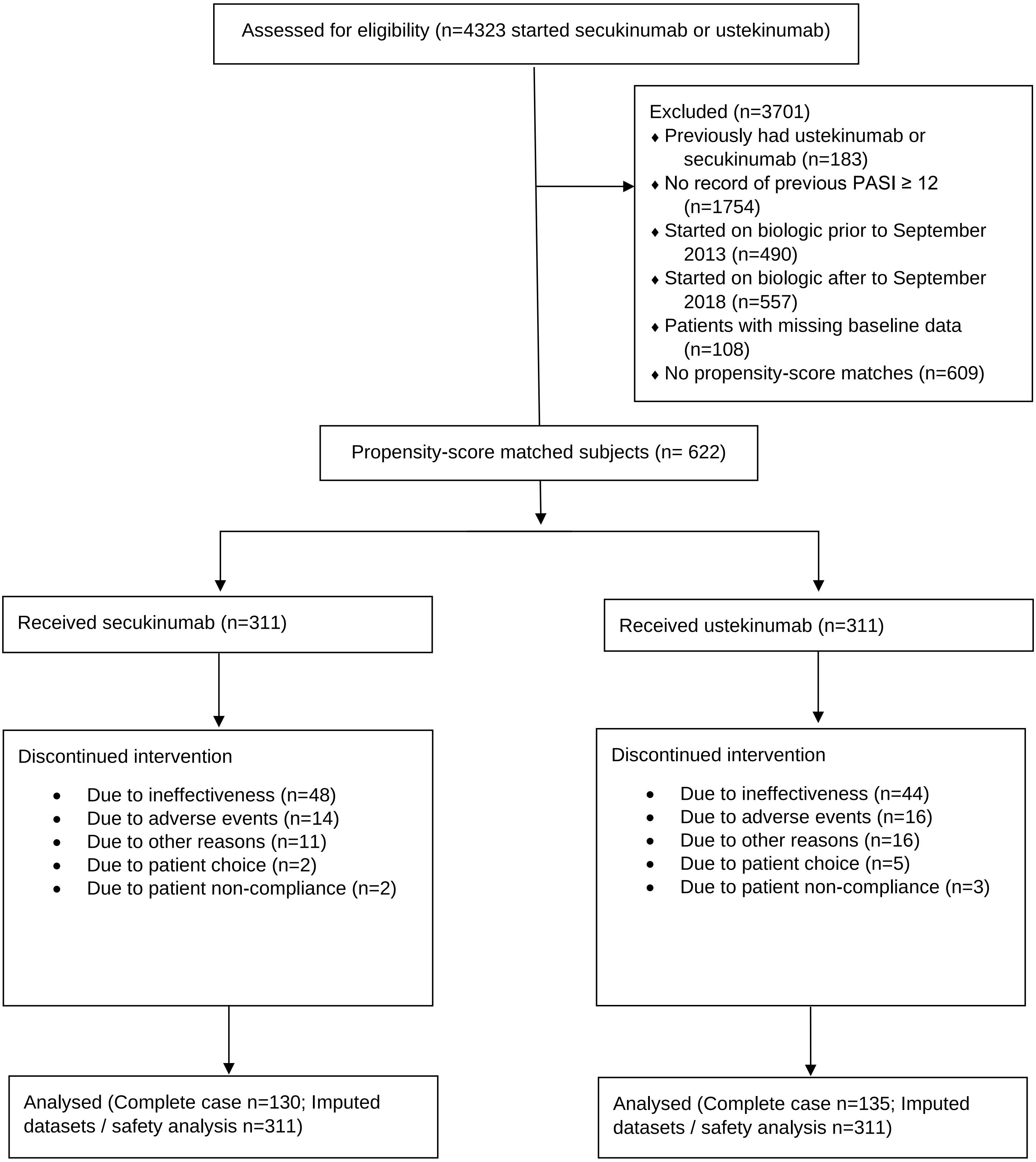

© 2020 Yiu ZZN et al. *JAMA Dermatology.*

eFigure 3. Forest Plot Summarising the Risk Difference Estimates for the Proportion of Participants Achieving PASI ≤ 2 at 12 Months Comparing Secukinumab Against Ustekinumab Using the Two Propensity Score Methods and the Missing Outcome Analysis Methods

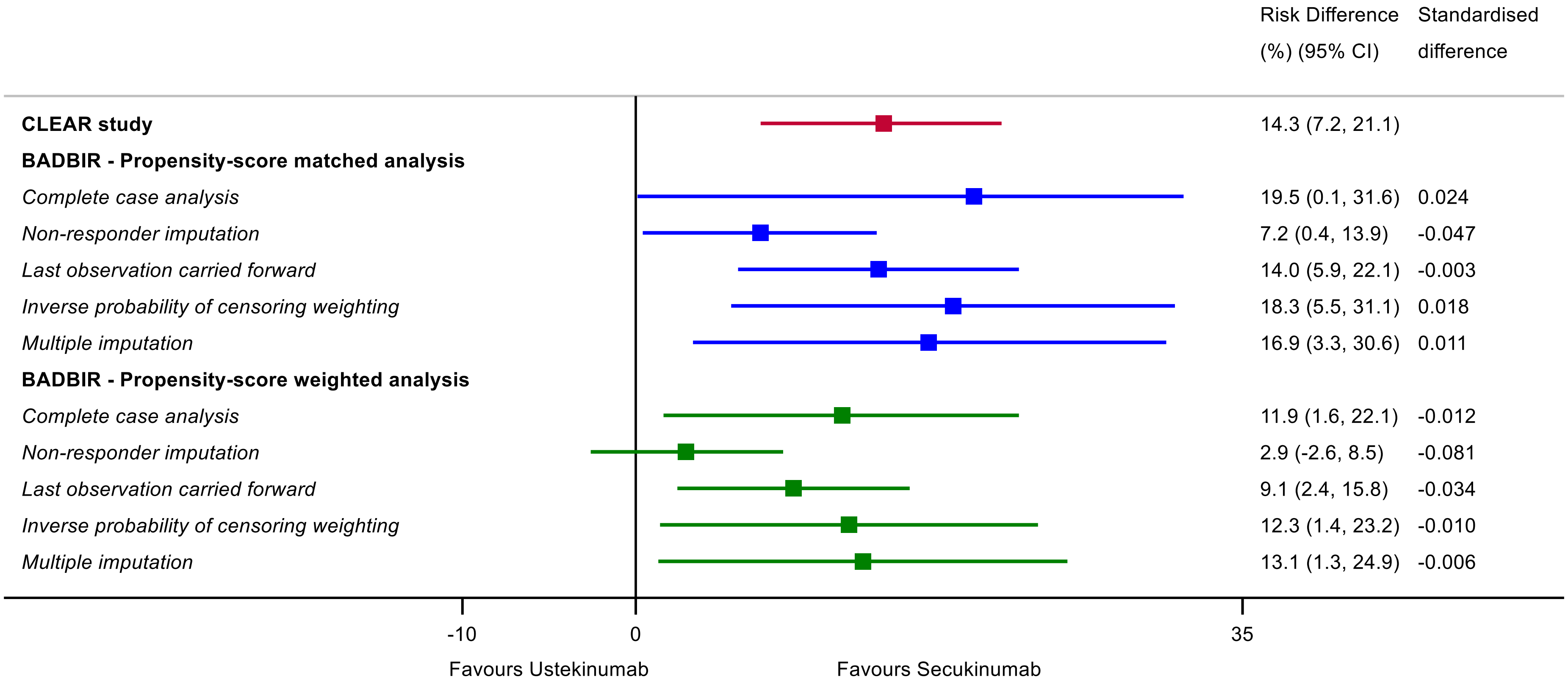

© 2020 Yiu ZZN et al. *JAMA Dermatology.*
